# Diagnostic Efficacy across Dense and Non-Dense Breasts during Digital Breast Tomosynthesis and Ultrasound Assessment for Recalled Women

**DOI:** 10.3390/diagnostics12061477

**Published:** 2022-06-16

**Authors:** Ibrahim Hadadi, Jillian Clarke, William Rae, Mark McEntee, Wendy Vincent, Ernest Ekpo

**Affiliations:** 1 Medical Image Optimisation and Perception Group, Discipline of Medical Imaging Science, Faculty of Medicine and Health, The University of Sydney, Camperdown, NSW 2006, Australia; jillian.clarke@sydney.edu.au (J.C.); will.rae@sydney.edu.au (W.R.); mark.mcentee@ucc.ie (M.M.); ernest.ekpo@sydney.edu.au (E.E.); 2 Department of Radiological Sciences, Faculty of Applied Medical Sciences, King Khalid University, Abha 62529, Saudi Arabia; 3 Medical Imaging Department, Prince of Wales Hospital, Sydney, NSW 2031, Australia; 4 Discipline of Diagnostic Radiography, UGF 12 ASSERT, Brookfield Health Sciences, University College Cork, College Road, T12 AK54 Cork, Ireland; 5 Royal Prince Alfred Hospital, Sydney Local Health District, Camperdown, NSW 2050, Australia; wendy.vincent@health.nsw.gov.au; 6 Orange Radiology, Laboratories and Research Centre, Calabar 540281, Nigeria

**Keywords:** ultrasound, DBT, breast density, breast cancer, equivocal (RANZCR 3)

## Abstract

Background: To compare the diagnostic efficacy of digital breast tomosynthesis (DBT) and ultrasound across breast densities in women recalled for assessment. Methods: A total of 482 women recalled for assessment from January 2017 to December 2019 were selected for the study. Women met the inclusion criteria if they had undergone DBT, ultrasound and had confirmed biopsy results. We calculated sensitivity, specificity, PPV, and AUC for DBT and ultrasound. Results: In dense breasts, DBT showed significantly higher sensitivity than ultrasound (98.2% vs. 80%; *p* < 0.001), but lower specificity (15.4% vs. 55%; *p* < 0.001), PPV (61.3% vs. 71%; *p* = 0.04) and AUC (0.568 vs. 0.671; *p* = 0.001). In non-dense breasts, DBT showed significantly higher sensitivity than ultrasound (99.2% vs. 84%; *p* < 0.001), but no differences in specificity (22% vs. 33%; *p* = 0.14), PPV (69.2% vs. 68.8%; *p* = 0.93) or AUC (0.606 vs. 0.583; *p* = 0.57). Around 73% (74% dense and 71% non-dense) and 77% (81% dense and 72% non-dense) of lesions assigned a RANZCR 3 by DBT and ultrasound, respectively, were benign. Conclusion: DBT has higher sensitivity, but lower specificity and PPV than ultrasound in women with dense breasts recalled for assessment. Most lesions rated RANZCR 3 on DBT and ultrasound are benign and may benefit from short interval follow-up rather than biopsy.

## 1. Introduction

Breast cancer screening using two-dimensional (2D) mammography is currently the primary standard of care [1]. In women aged 40–69 years, screening resulted in a significant—44%—reduction in breast cancer mortality, compared to 16% in those who were not screened [2]. The decline in mortality can be attributed to timely detection of breast cancer through screening and to developments in breast cancer management and treatment [3,4,5]. This drop in mortality is a major step toward minimising the breast cancer burden. Despite the benefits accrued through screening, limitations remain around the diagnostic accuracy of digital mammography (DM). Further, 2D mammography misses between 20% and 30% of breast cancers because of a masking effect in dense breast parenchyma, resulting in low sensitivity [6]. On the other hand, superimposition of normal fibroglandular tissue can yield erroneous mammograms, causing a high rate of unnecessary recalls [7,8].

Women with dense breasts are three to six times more likely to develop breast cancer than women with non-dense breasts [9,10]. Importantly, dense tissue also increases the risk of interval cancer due to the overall increased risk of cancer and the masking of lesions in dense areas of the breast [11]. To mitigate the limitations of DM, digital breast tomosynthesis (DBT) and ultrasound are used as adjuncts to mammography. These tools can reduce the anatomical noise that limits DM. Breast ultrasound is currently used primarily as an adjunct to assess suspicious findings identified during DM. It has been shown that ultrasound could detect small invasive cancers regardless of the density of a woman’s breast [12]. However, ultrasound has some limitations such as operator-dependence, low sensitivity in calcifications, a typical feature of ductal carcinoma in situ, and a high frequency of false positives [12,13]. There is limited evidence that ultrasound is sufficient to be used as an adjunct to DM in screening programs; however, the literature suggests that using ultrasound in conjunction with DM may be beneficial, particularly in women at high risk of developing breast cancer [14].

DBT is becoming more widely used, and there is growing evidence that it can significantly reduce false positive diagnosis when compared to DM alone. DBT minimises anatomical noise by creating pseudo cross-sectional images of the breast, which reduces the overlap of breast tissue, allowing for improved differentiation between normal and pathological tissues, and improving visualisation of lesions [15]. However, published studies show inconsistent data regarding recall rates of DBT. Studies conducted in the US showed that DBT was associated with a decrease in the recall rate by 1.5–3.8% [16,17,18,19,20]; however, European and Australian studies [21,22,23,24] found that DBT increased recall rates by 0.8–1.2%. These heterogeneous results could be explained by differences in the threshold used for recalling cases, study design and paired/unpaired reading in the screening settings where the studies were conducted. Other contributing factors include the experience of the readers in DBT [21,22,23] and the use of a one-view DBT [24].

Despite the widespread use of DBT and ultrasound tools, only a few studies [25,26,27] have compared DBT with ultrasound. Importantly, no study has directly compared the diagnostic efficacy of DBT and ultrasound in the assessment of mammography-recalled women, taking into consideration the density of a woman’s breast. Kim et al. [25] compared DBT and ultrasound for screening and diagnosis, the ASTOUND-2 trial [26] examined mammographically negative cases, and González-Huebra et al. [27] focused on preoperative lesions. Given that breast density is a major factor affecting the detection and characterisation of breast lesions on imaging, it is important to consider density when establishing the diagnostic efficacy of DBT and ultrasound in women recalled for assessment to better inform assessment pathways for women of different breast compositions. However, studies [25,26,27] that have compared the diagnostic performances of DBT and ultrasound for malignancies focused on lesions classified as suspicious (BI-RADS 4) or higher. In the Australian breast cancer screening program, the RANZCR scale is used to classify breast lesions. The RANZCR grading system demonstrates some differences with BI-RADS. For example, RANZCR grade 3 is a combination of BI-RADS 3 and BI-RADS 4A in the BI-RADS Atlas [28], and there is a paucity of data on the diagnostic efficacy of DBT and ultrasound in lesions rated RANZCR 3 on screening mammography. Identifying the assessment tools that best discriminate RANZCR 3 lesions as benign or malignant may improve practices and policies around the management of women with such lesions in Australia. Therefore, this study aims to compare the diagnostic efficacy of DBT and ultrasound across breast densities in women recalled for assessment. It also aims to evaluate the diagnostic efficacy of DBT and ultrasound in lesions rated RANZCR Grade 3 on DM.

## 2. Materials and Methods

### 2.1. Patient Selection

Within the Australian breast screening program, women recalled for assessment may undergo clinical breast examination, mammography spot views, DBT, ultrasound, and, if necessary, a percutaneous biopsy. For the current study, a total of 640 recalled women (mean age: 57; SD: ±8.6 years) were identified in the BreastScreen database. Women met the inclusion criteria if they had undergone both DBT and ultrasound examinations and had confirmed biopsy results (benign or malignant). Patients were excluded from the study if their specimens were not available or inadequate, had atypical/equivocal biopsy results (*n* = 144 patients), or if their breast density was not reported (*n* = 14 patients). After applying inclusion and exclusion criteria, data of 482 patients (mean age: 59.3; SD: ±8.8 years) were selected for the study. The demographic information of these patients is summarised in Table 1.

### 2.2. Study Design

We retrospectively reviewed the radiologists’ reports for the recalled women. In the BreastScreen program, the screening mammography cases are independently interpreted by two radiologists. All cases were interpreted by radiologists trained in mammography image interpretation and dedicated to breast imaging, and all were involved directly in the clinical and screening activities within the BreastScreen program. Cases independently rated as being suspicious of malignancy by two radiologists were recalled based on the RANZCR breast imaging lesion classification used by BreastScreen Australia [28]. This classification system is based on a simple 1–5 grading scale: 1 = ‘no significant abnormality’, 2 = ‘benign’, 3 = ‘equivocal’, 4 = ‘suspicious lesion’ and 5 = ‘malignant lesion’. Two mammographic views were acquired for each breast: cranio-caudal (CC) and medio-lateral oblique (MLO). Further mammography spot views were acquired if deemed necessary. The case is returned to routine screening if it is classified as no significant abnormality (RANZCR 1) or benign (RANZCR 2) but recalled for assessment if it is classified as equivocal (RANZCR 3), suspicious (RANZCR 4), or malignant (RANZCR 5). Cases rated RANZCR 3, 4, or 5 on screening mammography were assessed using DBT, ultrasound, and percutaneous needle biopsy (fine needle aspiration (FNA) cytology or core biopsy). Breast density was reported according to the Breast Imaging Reporting and Data System (BI-RADS, 5th edition) [29]:

BI-RADS A: “The breasts are almost entirely fatty”

BI-RADS B: “There are scattered areas of fibroglandular density”

BI-RADS C: “The breasts are heterogeneously dense, which may obscure small masses”

BI-RADS D: “The breasts are extremely dense, which lowers the sensitivity of mammography”

DBT and ultrasound examinations were performed before patients were referred for biopsies. DBT scanning was performed using Selenia Dimensions, Hologic. Radiologic features such as calcification, stellate lesions, discrete masses, non-specific density, architectural distortion, and multiple masses were used to describe lesions identified on DBT. Real-time B-mode Breast ultrasound was performed using an ACUSON S2000 Ultrasound System (HELX Evolution with Touch Control, Siemens Medical Solutions), equipped with a 12L4 linear array transducer (12–4 MHz). Colour Doppler was also used for characterisation of breast lesions. Where a lesion was detected on ultrasound, the sonographic features were described. The descriptions included indeterminate mass, cystic mass, solid mass (probably benign), solid mass (probably malignant) and axillary lymph nodes. Lesions detected on DBT and ultrasound were also rated using the RANZCR breast imaging lesion classification scale. Information such as lesion size, lesion location, tumour grade, patient age and personal/family history of breast cancer were retrieved from the database. Both DBT and ultrasound were interpreted by one radiologist depending on the digital mammographic findings.

### 2.3. Histopathological Testing

All cases graded as equivocal, suspicious, or malignant were biopsied using needle core biopsy or FNA with image guidance, e.g., ultrasound or mammography, as part of the BreastScreen Australia program. Needle core biopsy was the procedure of choice, while FNA was limited to simple cysts and lymph nodes. Needle core biopsy provided histological confirmation of malignant status (e.g., invasive or non-invasive), cancer type, and tumour grade in breast malignancies.

### 2.4. Statistical Analysis

Using these data, we calculated the diagnostic performance of DBT and ultrasound in terms of sensitivity, specificity, positive predictive value (PPV) and the area under the curve of the receiver operator characteristics (AUC) curve across dense and non-dense breasts. For the analysis, the RANZCR breast imaging lesion classifications of 1 and 2 were considered as negative findings, and classifications of 3, 4, and 5 were considered positive findings. For breast density, cases categorised as BI-RADS A and B were considered non-dense breasts, and those classified as BI-RADS C and D were considered dense breasts. The difference between cancer sizes in dense and non-dense breasts were compared using a Mann–Whitney U test. McNemar’s test was used to compare the sensitivity and specificity of DBT and ultrasound in dense and non-dense breasts. The Two Proportion Z-Test was used to compare the PPVs of DBT and ultrasound in dense and non-dense breasts. The method for paired sample design, devised by Delong et al. [30] was employed to compare the AUCs of DBT and ultrasound in dense and non-dense breasts. A *p*-value ≤ 0.05 was considered statistically significant. These statistical analyses were conducted via the open-source Jamovi software (1.6.22) and R statistical software (4.0.3).

## 3. Results

**Figure 1:**
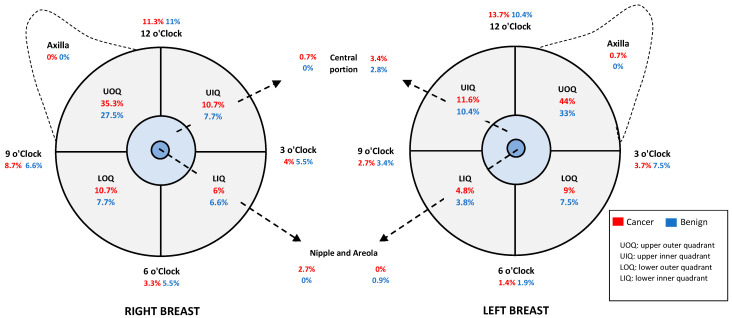
The distribution of breast lesion locations in the right and left breast.

A total of 492 breast lesions from 482 women (mean age: 59.3, SD: ±8.8 years; range: 40–94 years), who received both DBT and ultrasound followed by histopathological tests, were examined. The needle biopsy revealed 296 breast cancers (232 invasive, 64 non-invasive) and 197 benign lesions. Approximately 38% of invasive breast cancers detected were Grade 2. The sizes of breast cancers significantly differed between dense and non-dense breasts (*p* < 0.001). In dense breasts, the median size of the breast cancers was 1.4 cm (range: 0.4–10 cm), with 34.3% of the cancers ≤1 cm. In non-dense breasts, the median size of the breast cancers was 1.1cm (range: 0.3–13 cm); 49% of breast cancers were ≤1 cm. Table 2 summarises breast cancer characteristics in relation to breast density. Most breast lesions were localised in the upper outer quadrants (UOQs) of the right and left breast; nearly 40% were malignant, and 31% were benign. Figure 1 shows the distribution of breast lesion locations.

Table 3 shows the diagnostic performance of DBT and ultrasound across dense and non-dense breasts for recalled cases. In dense breasts, the sensitivity of DBT was 98.2% (95% CI: 94.8–99.6), significantly higher than that of ultrasound (80%; 95% CI: 73–85.6; *p* < 0.001). The specificity of DBT was 15.4% (95% CI, 9.6–23), significantly lower than that of ultrasound (55%; 95% CI: 44.8–63; *p* < 0.001). The PPVs of DBT was 61.3% (95% CI, 55.2–67), significantly lower than that of ultrasound (71%; 95% CI: 64–77; *p* = 0.04). DBT poorly discriminated between malignant and benign lesions (AUC: 0.568; 95% CI: 0.501–0.636), significantly lower than the discriminatory power of ultrasound, which was 0.671 (95% CI: 0.607–0.735), (*p* = 0.001).

For women recalled due to the presence of calcification(s) (*n* = 107) in their mammograms, the sensitivity of DBT was 100% (95% CI: 93.4–100), significantly higher than that of ultrasound (37%; 95% CI: 24.3–51.3; *p* < 0.001). The specificity of ultrasound was 92.5% (95% CI: 82–98), significantly higher than DBT (2%; 95% CI, 0–10.1; *p* < 0.001). The PPVs of DBT was 51% (95% CI, 41–61), significantly lower than that of ultrasound (83.3%; 95% CI: 63–95.3; *p* = 0.003). Ultrasound performed better in discriminating between calcified benign and malignant lesions (AUC: 0.647; 95% CI: 0.543–0.752) than DBT (AUC: 0.509; 95% CI: 0.400–0.619, *p* < 0.001).

For women recalled due to other radiologic features (*n* = 183) (e.g., stellate, discrete mass, non-specific density or architectural distortion), DBT and ultrasound achieved similar performance in sensitivity (97.3%; 95% CI: 92.3–99 vs. 100%; 95% CI: 96.7–100; *p* = 0.08), specificity (25%; 95% CI: 15.5–36 vs. 24%; 95% CI: 14.4–35.1; *p* = 0.8), PPVs (67%; 95% CI: 58.8–73.9 vs. 70%; 95% CI: 59.2–74; *p* = 0.97) and AUCs (0.611; 95% CI: 0.525–0.698 vs. 0.618; 95% CI: 0.531–0.705; *p* = 0.82).

In non-dense breasts, there were no significant differences between DBT and ultrasound in specificity (23%; 95% CI: 13.1–33.1 vs. 33%; 95% CI: 22.3–45; *p* = 0.14), PPV (69.2%; 95% CI: 62–76 vs. 68.8%; 95% CI: 61–75.9; *p* = 0.93) or AUC (0.606; 95% CI: 0.521–0.691 vs. 0.583; 95% CI: 0.499–0.667; *p* = 0.57). However, the sensitivity of DBT was significantly higher than that of ultrasound (99.2%; 95% CI: 95.8–100 vs. 84%; 95% CI: 76.2–90; *p* < 0.001).

For women recalled due to the presence of calcification in their mammograms (*n* = 31), the sensitivity of DBT was 100% (95% CI: 75.3–100), significantly higher than that of ultrasound (54%; 95% CI: 25–81; *p* = 0.008). The specificity of DBT was 0% (95% CI, 0–19), significantly lower than that of ultrasound (72.2%; 95% CI: 47–90; *p* = 0.02). There were no significant differences between DBT and ultrasound in PPV (42%; 95% CI: 42–42 vs. 58.3%; 95% CI: 29–85; *p* = 0.33) or AUC (0.500; 95% CI: 0.291–0.709 vs. 0.630; 95% CI: 0.427–0.834; *p* = 0.15).

For women recalled due to other radiologic features (*n* = 171), DBT also achieved significantly higher sensitivity than ultrasound (99%; 95% CI: 95.3–100 vs. 87%; 95% CI: 80–93; *p* < 0.001). However, the specificity of DBT was 16.3% (95% CI, 8–29), significantly lower than that of ultrasound (33%; 95% CI: 21–46.7; *p* = 0.03). There were no significant differences between DBT and ultrasound in PPV (71.4%; 95% CI: 64–78.3 vs. 73.2%; 95% CI: 65–80.4; *p* = 0.72) or AUC (0.577; 95% CI: 0.480–0.673 vs. 0.599; 95% CI: 0.504–0.694; *p* = 0.58).

Table 4 summarises the performance of DBT and ultrasound in the evaluation of breast lesions classified as RANZCR 3 on DM across dense and non-dense breasts.

## 4. Discussion

Even with excellent mammographic technique and independent double reading, an image may be difficult to interpret, lesion may not be well categorised, or radiologists may show inter-reader disagreement. Assessment modalities, such as DBT and ultrasound, may be required to thoroughly evaluate recalled mammographic findings. Both techniques, however, may differ in their classification of breast lesion types. In this study, we compared the diagnostic performance of DBT and ultrasound in women with dense and non-dense breasts. We found that, in dense breasts, DBT showed significantly higher sensitivity, but significantly lower specificity, PPV and AUC, than ultrasound. In non-dense breasts, the sensitivity of DBT was also significantly higher than that of ultrasound; however, no significant differences were found in specificity, PPV or AUC.

Our findings differ from previous studies [25,26,27] due to the following reasons. First, it is the first to compare DBT and ultrasound in mammography-recalled women across dense and non-dense breasts. Second, the synoptic breast imaging report used by BreastScreen Australia differs from the interpretation strategies used in non-Australian studies. A score of 3 in the US BI-RADS lexicon indicates that the lesion will require a six-month follow-up, whereas a score of 3 in the RANZCR scoring scheme indicates further assessment and biopsy, which may increase unnecessary recalls. Third, our data contains many recalled calcifications, which remain a challenge for ultrasound [31,32].

Our findings that adding DBT and ultrasound to screening programs enhances the early detection of breast cancers in both dense and non-dense breasts are consistent with the literature [12,33]. Importantly, we found that cancers detected by DBT, and ultrasound are small and/or invasive DBT and ultrasound detected many breast cancers, including 79% of invasive cancers and 49% of cancers smaller than 1 cm. Early cancer detection for small, invasive cancers is beneficial for prognosis and treatment [6,34,35]. It has been reported that the 10-year survival from breast cancer is substantially higher for women with small-sized cancers. For example, the 10-year survival from breast cancers no larger than 1 cm is 87%, compared to 76% and 75% for cancers ranging in size from 1.1–2 cm and 2.1–3 cm, respectively [36]. Therefore, imaging tools that improve the detection of small-sized malignant tumours could improve treatment outcome. Another interesting finding with respect to tumour size was that cancers detected in dense breasts were significantly larger than those in non-dense breasts. Larger sized tumours in dense breasts can be explained by two factors. Firstly, dense tissue contains high proportion of stromal cells, which regulate the proliferation of epithelial cells and are progenitors of collagen, which binds to growth hormone to support tumour reorganisation. These factors may act together to facilitate rapid growth of tumours in dense compared to fatty breasts. Secondly, some of the lesions in dense tissue may be interval cancers or cancers that were missed at previous mammography screening due to the masking effect of mammographic density [37,38,39]. Regardless, the findings suggest the need to tailor screening intervals and pathways according to mammographic density to detect small-sized early-stage disease.

We observed that calcifications were more likely to be classified as positive on DBT relative to ultrasound in all breast compositions. This is indicated by the significantly higher sensitivity on DBT compared to ultrasound. Nevertheless, the advantages of DBT in detecting calcifications (100% sensitivity) should be balanced against its disadvantages, which include low specificity and PPV, particularly in dense breasts. Given the high prevalence of calcifications in the screening population, reasonable positive thresholds are required to increase both specificity and PPV for DBT. A previous work shows that DBT improves diagnostic accuracy in suspicious calcification features, with an excellent AUC of 0.903 in dense breasts and 0.904 in non-dense breasts [40]. We found that DBT shows very low AUCs in suspicious calcifications, for both dense (0.509) and non-dense breasts (0.500). It should be noted that the previous work included only women who had a biopsy for suspicious calcifications (BI-RADS 4A or higher), and classified BI-RADS 4A as a negative finding since it indicates a low risk of malignancy according to the BI-RADS Atlas. These differences in study methods could have influenced the results [40].

Furthermore, we observed that ultrasound underestimated most calcification features, classifying them as RANZCR grade 1. This RANZCR rating suggests that the calcifications may have been missed or dismissed on ultrasound, particularly in dense breasts, where the sensitivity was only 37%. It has been shown that Cooper’s ligaments and ductal walls may mimic calcifications, particularly in fibrocystic changes [41,42], and may be responsible for calcifications being dismissed. It should also be noted that the efficacy of ultrasound is dependent on the operator’s experience and the transducer technology. For example, a 7.5 MHz transducer has a lateral resolution of approximately 1 mm, while calcifications typically measure 0.1–1.0 mm. Therefore, ultrasound could miss calcifications smaller than 1 mm [42]. The inverse relationship between transducer frequency and beam penetration may have also contributed to the low sensitivity of ultrasound for calcification in dense breasts [32]. Our findings suggest that mammography-recalled calcifications should not be completely ruled out solely on ultrasound findings.

In noncalcified lesions, DBT was comparable to ultrasound in dense breasts, but showed significantly higher sensitivity in non-dense breasts. Noncalcified lesions are mostly hypoechoic; the contrast between hypoechoic tumour and echogenic dense tissue may contribute to the high sensitivity of ultrasound in dense breasts [43,44]. The high sensitivity of DBT in women recalled suggests that 2D DM spot views may not be needed where this assessment tool is available. This is further supported by the findings that, in all breast compositions, the RANZCR grades assigned to the additional spot views and the DBT assessments were in significant agreement, as shown in Supplementary Materials (Table S1).

Recalling lesions with a low probability of cancer [28] using the RANZCR Grade 3 (a combination of BI-RADS 3/4A) may be justifiable for the following reasons. First, the screening program’s primary purpose is to detect cancers in their earliest stages [45]. Second, DM is also affected by breast density, prompting the use of assessment tools to optimise breast cancer diagnosis. However, our findings show that using the same biopsy threshold for both DBT and ultrasound should be reconsidered because reducing false positives is just as important as improving breast cancer detection [7]. Our findings on DBT are consistent with a recent Australian study [21], which reported improved cancer detection rates but increased false positives. The high number of false positives could be caused by excessive use of the RANZCR Grade 3 in Australia. It is possible that the high false-positive rate associated with DBT may decrease in Australia as screening program readers gain expertise with this technology [21,46].

**Figure 2:**
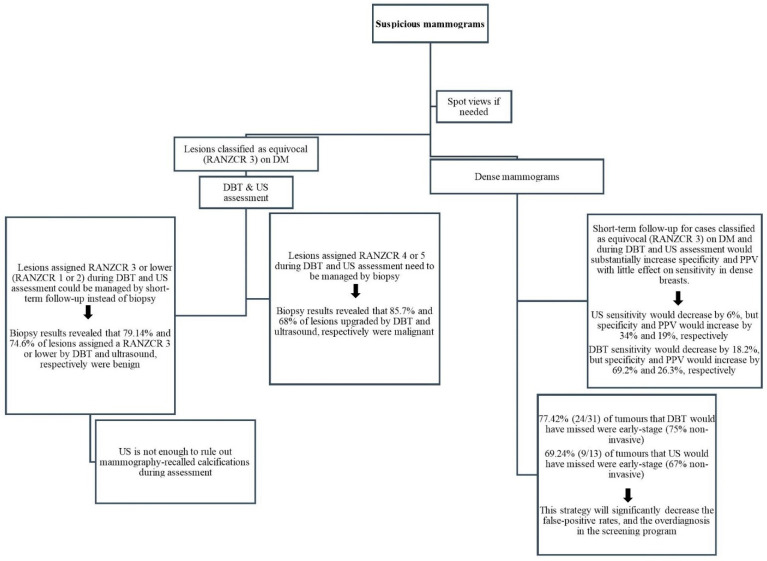
Proposed system architecture for optimising breast cancer detection during DBT and US assessment.

We found that DBT changed lesion classification in 36% of cases rated RANZCR 3 on DM, with an upgrade in 22% (86% of which had breast cancer) and a downgrade in 14% (90% of which were benign). Whereas ultrasound changed the classification in 71% of lesions rated RANZCR 3 on DM, with an upgrade in 29% (68% of which had breast cancer) and a downgrade in 42% (72% of which were benign). Another interesting finding was that the benign rate of lesions where DBT and ultrasound led to no change in classification (RANZCR 3) was much higher than the cancer rate. Biopsy results revealed that around 73% (74% dense and 71% non-dense) and 77% (81% dense and 72% non-dense) of lesions assigned a RANZCR 3 by DBT and ultrasound, respectively, were benign (Table 4). Thus, considering short-term follow-up instead of biopsy for cases classified as equivocal (RANZCR 3) on DM and during DBT and US assessment may reduce unnecessary biopsies, particularly in dense breasts (Figure 2). This strategy may lead to a small number of invasive cancers being missed, but will significantly decrease the false-positive rates, as well as decrease the overdiagnosis in the screening program. Previous studies [47,48,49] show that low-risk category ratings (BI-RADS 3/4A/4B) safely decreased the rate of biopsies for false-positive results. In addition, cancers originally classified as low-risk were found to be early-stage tumours when biopsies were conducted during a short follow-up or at a subsequent screening; this did not lead to clinically significant delays in breast cancer diagnosis. These findings suggest that the use of the RANZCR Grade 3 during DBT and ultrasound assessments in Australia should be reconsidered, as should views regarding short interval follow-ups. Reconsidering the use of RANZCR grade 3 may provide a baseline for identifying what thresholds will optimise cancer detection, while minimising unnecessary biopsies.

This study is not without limitations. All radiologists had prior knowledge of the original mammographic findings when interpreting subsequent DBT and ultrasound images. This factor could influence the radiologists’ decision-making, leading to the increased false-positive rates for both modalities. Furthermore, our data is from a single centre. Larger multi-centre studies are needed to verify and translate the results of our study. Conversely, our data represents real-life clinical experience of using DBT and ultrasound for assessment of mammography recalled women and account for the independent double reading system practiced in Australia, which has been associated with increased breast cancer detection [50]. To our knowledge, this is the first study that has compared the diagnostic performance of DBT and ultrasound in women recalled due to digital mammographic findings in BreastScreen Australia. Therefore, our study provides baseline data to inform assessment modalities of women recalled for additional imaging due to mammography findings.

## 5. Conclusions

Digital breast tomosynthesis has higher sensitivity, but lower specificity and positive predictive value than ultrasound in women with dense breasts recalled for assessment. Both DBT and ultrasound demonstrate significant limitations in the assessments of calcifications, with DBT limited in correctly characterising benign calcifications and ultrasound underestimating the malignant potential of many malignant calcifications. Most lesions rated RANZCR Grade 3 on DBT and ultrasound assessments are benign and may benefit from short interval follow-up rather than biopsy. Therefore, optimising the assessment of calcifications and lesions rated RANZCR 3 as well as the thresholds for biopsy recommendations for these lesions may reduce unnecessary biopsies and improve the management of women with such lesions.

## Figures and Tables

**Table 1:** Patients’ demographic information.

Characteristic	Patients No. (%)
Age (years)	
40–49	60 (12.4)
50–59	188 (39)
60–69	156 (32.4)
70–79	74 (15.4)
≥80	4 (0.8)
Breast Density	
Almost entirely fatty	58 (12)
Scattered areas of fibroglandular density	142 (29.4)
Heterogeneously dense	206 (42.8)
Extremely dense	76 (15.8)
Risk Factor	
Personal history of Breast Cancer	8 (1.67)
Family History of Breast Cancer	78 (16.2)
Personal and family history of Breast Cancer	4 (0.83)
No personal and family history of Breast Cancer	392 (81.3)

**Table 2:** Breast cancer characteristics across dense and non-dense breasts.

Characteristic	Dense Breasts No. (%)	Non-Dense Breasts No. (%)
Cancer Type		
Malignant–Invasive	121 (72.5)	111 (86)
Malignant–Non-invasive	46 (27.5)	18 (14)
Tumor Grade		
Grade 1	34 (20.5)	38 (29.4)
Grade 2	63 (38)	49 (38)
Grade 3	24 (14)	26 (20.2)
Not specified	46 (27.7)	16 (12.4)
Size		
≤1 cm	59 (35)	63 (48.9)
1.1–2 cm	56 (33.8)	49 (38)
2.1–3 cm	26 (15.6)	10 (7.7)
>3 cm	26 (15.6)	7 (5.4)

Dense Breast: BI-RADS C & D; Non-Dense Breast: BI-RADS A & B.

**Table 3:** Diagnostic performance of DBT and ultrasound across dense and non-dense breasts.

Breast Density	Modality	Sensitivity (95% CI)	*p*	Specificity (95% CI)	*p*	PPV (95% CI)	*p*	ROC AUC (95% CI)	*p*
**Dense Breasts**									
**All cases**	DBT	98.2 (94.8–99.6)	**<0.001**	15.4 (9.6–23)	**<0.001**	61.3 (55.2–67)	**0.04**	0.568 (0.501–0.636)	**0.001**
	US	80 (73–85.6)		55 (44.8–63)		71 (64–77)		0.671 (0.607–0.735)	
Calcifications Feature **present**	DBT	100 (93.4–100)	**<0.001**	2 (0–10.1)	**<0.001**	51 (41–61)	**0.003**	0.509 (0.400–0.619)	**<0.001**
	US	37 (24.3–51.3)		92.5 (82–98)		83.3 (63–95.3)		0.647 (0.543–0.752)	
**none**	DBT	97.3 (92.3–99)	0.08	25 (15.5–36)	0.8	67 (58.8–73.9)	0.97	0.611 (0.525–0.698)	0.82
	US	100 (96.7–100)		24 (14.4–35.1)		70 (59.2–74)		0.618 (0.531–0.705)	
**Non-Dense Breasts**									
All cases	DBT	99.2 (95.8–100)	**<0.001**	22 (13.1–33.1)	0.14	69.2 (62–76)	0.93	0.606 (0.521–0.691)	0.57
	US	84 (76.2–90)		33 (22.3–45)		68.8 (61–75.9)		0.583 (0.499–0.667)	
Calcifications Feature **present**	DBT	100 (75.3–100)	**0.008**	0 (0–19)	**0.02**	42 (42–42)	0.33	0.500 (0.291–0.709)	0.15
	US	54 (25–81)		72.2 (47–90)		58.3 (29–85)		0.630 (0.427–0.834)	
**none**	DBT	99 (95.3–100)	**<0.001**	16.4 (8–29)	**0.03**	71.4 (64–78.3)	0.72	0.577 (0.480–0.673)	0.58
	US	87 (80–93)		33 (21–46.7)		73.2 (65–80.4)		0.599 (0.504–0.694)	

Dense Breast: BI-RADS C & D; Non-Dense Breast: BI-RADS A & B; DBT: digital breast tomosynthesis; US: ultrasound; *p* value for McNemar’s test comparing between sensitivity and specificity values of DBT and US across dense and non-dense breasts; AUC values of DBT and US were compared using the method of Delong et al. (1988) for paired sample design. PPVs were compared using Two Proportion Z-Test.

**Table 4:** The performance of DBT and ultrasound in the categorisation of 284 lesions classified as equivocal (RANZCR 3) on DM.

**DBT**						
**Biopsy Results**	**Breast Density**	**No Significant Abnormality**	**Benign Lesion**	**Equivocal Lesion**	**Suspicious Lesion**	**Malignant Lesion**
Benign	Dense	5	14	85	5	1
	Non-Dense	2	13	48	3	0
Non-Invasive Cancer	Dense	0	0	18	6	1
	Non-Dense	0	1	6	3	0
Invasive Cancer	Dense	1	2	12	14	10
	Non-Dense	0	0	14	17	3
**US**						
**Biopsy Results**	**Breast Density**	**No Significant Abnormality**	**Benign Lesion**	**Equivocal Lesion**	**Suspicious Lesion**	**Malignant Lesion**
Benign	Dense	52	11	42	4	1
	Non-Dense	16	6	23	9	12
Non-Invasive Cancer	Dense	19	1	3	1	1
	Non-Dense	3	0	3	2	2
Invasive Cancer	Dense	4	1	7	12	15
	Non-Dense	6	0	6	12	10

Dense Breast: BI-RADS C & D; Non-Dense Breast: BI-RADS A & B; DBT: digital breast tomosynthesis; US: ultrasound.

## Data Availability

Data are available from the authors with the permission of BreastScreen NSW, Sydney Local Health District.

## Supplementary Materials

The following supporting information can be downloaded at: https://www.mdpi.com/article/10.3390/diagnostics12061477/s1, Table S1: The agreement of RANZCR breast lesion classifications between DBT and DM spot views on 219 lesions.

## References

[1] Nelson H.D., Fu R., Cantor A., Pappas M., Daeges M., Humphrey L. (2016). Effectiveness of breast cancer screening: Systematic review and meta-analysis to update the 2009 US Preventive Services Task Force recommendation. Ann. Intern. Med..

[2] Tabar L., Yen M.-F., Vitak B., Chen H.-H.T., Smith R.A., Duffy S.W. (2003). Mammography service screening and mortality in breast cancer patients: 20-year follow-up before and after introduction of screening. Lancet.

[3] Australian Institute of Health and Welfare (2012). Breast Screen Australia Monitoring Report 2009–2010.

[4] Rivera-Franco M.M., Leon-Rodriguez E. (2018). Delays in Breast Cancer Detection and Treatment in Developing Countries. Breast Cancer Basic Clin. Res.

[5] Herrmann C., Vounatsou P., Thürlimann B., Probst-Hensch N., Rothermundt C., Ess S. (2018). Impact of mammography screening programmes on breast cancer mortality in Switzerland, a country with different regional screening policies. BMJ Open.

[6] Kolb T.M., Lichy J., Newhouse J.H. (2002). Comparison of the performance of screening mammography, physical examination, and breast US and evaluation of factors that influence them: An analysis of 27,825 patient evaluations. Radiology.

[7] Skaane P. (2019). How Can We Reduce Unnecessary Procedures after Screening Mammography?. Radiology.

[8] Carbonaro L.A., Di Leo G., Clauser P., Trimboli R.M., Verardi N., Fedeli M.P., Girometti R., Tafà A., Bruscoli P., Saguatti G. (2016). Impact on the recall rate of digital breast tomosynthesis as an adjunct to digital mammography in the screening setting. A double reading experience and review of the literature. Eur. J. Radiol..

[9] Boyd N.F., Martin L.J., Bronskill M., Yaffe M.J., Duric N., Minkin S. (2010). Breast tissue composition and susceptibility to breast cancer. J. Natl. Cancer Inst..

[10] McCormack V.A., dos Santos Silva I. (2006). Breast density and parenchymal patterns as markers of breast cancer risk: A meta-analysis. Cancer Epidemiol. Prev. Biomark..

[11] Boyd N.F., Guo H., Martin L.J., Sun L., Stone J., Fishell E., Jong R.A., Hislop G., Chiarelli A., Minkin S. (2007). Mammographic Density and the Risk and Detection of Breast Cancer. N. Engl. J. Med..

[12] Hadadi I., Rae W., Clarke J., McEntee M., Ekpo E. (2021). Diagnostic Performance of Adjunctive Imaging Modalities Compared to Mammography Alone in Women with Non-Dense and Dense Breasts: A Systematic Review and Meta-Analysis. Clin. Breast Cancer.

[13] Moon W.K., Myung J.S., Lee Y.J., Park I.A., Noh D.-Y., Im J.-G. (2002). US of Ductal Carcinoma In Situ. RadioGraphics.

[14] Wang Y., Chen H., Li N., Ren J., Zhang K., Dai M., He J. (2019). Ultrasound for Breast Cancer Screening in High-Risk Women: Results from a Population-Based Cancer Screening Program in China. Front. Oncol..

[15] Houssami N., Skaane P. (2013). Overview of the evidence on digital breast tomosynthesis in breast cancer detection. Breast.

[16] Lourenco A.P., Barry-Brooks M., Baird G.L., Tuttle A., Mainiero M.B. (2015). Changes in Recall Type and Patient Treatment Following Implementation of Screening Digital Breast Tomosynthesis. Radiology.

[17] Hakim C.M., Catullo V.J., Chough D.M., Ganott M.A., Kelly A.E., Shinde D.D., Sumkin J.H., Wallace L.P., Bandos A.I., Gur D. (2015). Effect of the Availability of Prior Full-Field Digital Mammography and Digital Breast Tomosynthesis Images on the Interpretation of Mammograms. Radiology.

[18] Rose S.L., Tidwell A.L., Bujnoch L.J., Kushwaha A.C., Nordmann A.S., Sexton R. (2013). Implementation of Breast Tomosynthesis in a Routine Screening Practice: An Observational Study. Am. J. Roentgenol..

[19] McCarthy A.M., Kontos D., Synnestvedt M., Tan K.S., Heitjan D.F., Schnall M., Conant E.F. (2014). Screening Outcomes Following Implementation of Digital Breast Tomosynthesis in a General-Population Screening Program. JNCI J. Natl. Cancer Inst..

[20] Sprague B.L., Coley R.Y., Kerlikowske K., Rauscher G.H., Henderson L.M., Onega T., Lee C.I., Herschorn S.D., Tosteson A.N., Miglioretti D.L. (2020). Assessment of Radiologist Performance in Breast Cancer Screening Using Digital Breast Tomosynthesis vs Digital Mammography. JAMA Netw. Open.

[21] Houssami N., Lockie D., Clemson M., Pridmore V., Taylor D., Marr G., Evans J., Macaskill P. (2019). Pilot trial of digital breast tomosynthesis (3D mammography) for population-based screening in BreastScreen Victoria. Med. J. Aust..

[22] Bernardi D., Macaskill P., Pellegrini M., Valentini M., Fantò C., Ostillio L., Tuttobene P., Luparia A., Houssami N. (2016). Breast cancer screening with tomosynthesis (3D mammography) with acquired or synthetic 2D mammography compared with 2D mammography alone (STORM-2): A population-based prospective study. Lancet Oncol..

[23] Skaane P., Bandos A.I., Gullien R., Eben E.B., Ekseth U., Haakenaasen U., Izadi M., Jebsen I.N., Jahr G., Krager M. (2013). Comparison of Digital Mammography Alone and Digital Mammography Plus Tomosynthesis in a Population-based Screening Program. Radiology.

[24] Lång K., Andersson I., Rosso A., Tingberg A., Timberg P., Zackrisson S. (2016). Performance of one-view breast tomosynthesis as a stand-alone breast cancer screening modality: Results from the Malmö Breast Tomosynthesis Screening Trial, a population-based study. Eur. Radiol..

[25] Kim W.H., Chang J.M., Lee J., Chu A.J., Seo M., Gweon H.M., Koo H.R., Lee S.H., Cho N., Bae M.S. (2017). Diagnostic performance of tomosynthesis and breast ultrasonography in women with dense breasts: A prospective comparison study. Breast Cancer Res. Treat..

[26] Tagliafico A.S., Mariscotti G., Valdora F., Durando M., Nori J., La Forgia D., Rosenberg I., Caumo F., Gandolfo N., Sormani M.P. (2018). A prospective comparative trial of adjunct screening with tomosynthesis or ultrasound in women with mammography-negative dense breasts (ASTOUND-2). Eur. J. Cancer.

[27] González-Huebra I., Elizalde A., García-Baizán A., Calvo M., Ezponda A., Martínez-Regueira F., Pina L. (2019). Is it worth to perform preoperative MRI for breast cancer after mammography, tomosynthesis and ultrasound?. Magn. Reson. Imaging.

[28] The Royal Australian and New Zealand College of Radiologists Breast Imaging Grading Comparison and Lesion Classification Lists. https://www.ranzcr.com/college/document-library/breast-imaging-grading-comparison-and-lesion-classification.

[29] Sickles E., d’Orsi C., Bassett L., Appleton C., Berg W., Burnside E. (2013). Acr Bi-Rads^®^ Mammography. ACR BI-RADS^®^ Atlas, Breast Imaging Reporting and Data System.

[30] DeLong E.R., DeLong D.M., Clarke-Pearson D.L. (1988). Comparing the areas under two or more correlated receiver operating characteristic curves: A nonparametric approach. Biometrics.

[31] Demetri-Lewis A., Slanetz P.J., Eisenberg R.L. (2012). Breast calcifications: The focal group. Am. J. Roentgenol..

[32] Hooley R.J., Scoutt L.M., Philpotts L.E. (2013). Breast Ultrasonography: State of the Art. Radiology.

[33] Vourtsis A., Berg W.A. (2019). Breast density implications and supplemental screening. Eur. Radiol..

[34] Yun S.J., Ryu C.-W., Rhee S.J., Ryu J.K., Oh J.Y. (2017). Benefit of adding digital breast tomosynthesis to digital mammography for breast cancer screening focused on cancer characteristics: A meta-analysis. Breast Cancer Res. Treat..

[35] Kang D.K., Jeon G.S., Yim H., Jung Y.S. (2007). Diagnosis of the intraductal component of invasive breast cancer: Assessment with mammography and sonography. J. Ultrasound Med..

[36] Rosen P.P., Groshen S., Saigo P.E., Kinne D.W., Hellman S. (1989). Pathological prognostic factors in stage I (T1N0M0) and stage II (T1N1M0) breast carcinoma: A study of 644 patients with median follow-up of 18 years. J. Clin. Oncol..

[37] Roubidoux M.A., Bailey J.E., Wray L.A., Helvie M.A. (2004). Invasive Cancers Detected after Breast Cancer Screening Yielded a Negative Result: Relationship of Mammographic Density to Tumor Prognostic Factors. Radiology.

[38] Birdwell R.L., Ikeda D.M., O’Shaughnessy K.F., Sickles E.A. (2001). Mammographic Characteristics of 115 Missed Cancers Later Detected with Screening Mammography and the Potential Utility of Computer-aided Detection. Radiology.

[39] Conklin M.W., Keely P.J. (2012). Why the stroma matters in breast cancer: Insights into breast cancer patient outcomes through the examination of stromal biomarkers. Cell Adhes. Migr..

[40] Li J., Zhang H., Jiang H., Guo X., Zhang Y., Qi D., Guan J., Liu Z., Wu E., Luo S. (2019). Diagnostic performance of digital breast tomosynthesis for breast suspicious calcifications from various populations: A comparison with full-field digital mammography. Comput. Struct. Biotechnol. J..

[41] Nagashima T., Hashimoto H., Oshida K., Nakano S., Tanabe N., Nikaido T., Koda K., Miyazaki M. (2005). Ultrasound demonstration of mammographically detected microcalcifications in patients with ductal carcinoma in situ of the breast. Breast Cancer.

[42] Gufler H., Hernando Buitrago-Téllez C., Madjar H., Allmann K.H., Uhl M., Rohr-Reyes A. (2000). Ultrasound demonstration of mammographically detected microcalcifications. Acta Radiol..

[43] García-Barquín P., Páramo M., Elizalde A., Pina L., Etxano J., Fernandez-Montero A., Caballeros M. (2017). The effect of the amount of peritumoral adipose tissue in the detection of additional tumors with digital breast tomosynthesis and ultrasound. Acta Radiol..

[44] Stavros A. (2004). Benign solid nodules: Specific pathologic diagnosis. Breast Ultrasound.

[45] Tabár L., Vitak B., Chen T.H.-H., Yen A.M.-F., Cohen A., Tot T., Chiu S.Y.-H., Chen S.L.-S., Fann J.C.-Y., Rosell J. (2011). Swedish two-county trial: Impact of mammographic screening on breast cancer mortality during 3 decades. Radiology.

[46] Hadadi I., Rae W., Clarke J., McEntee M., Ekpo E. (2021). Breast cancer detection: Comparison of digital mammography and digital breast tomosynthesis across non-dense and dense breasts. Radiography.

[47] Baum J.K., Hanna L.G., Acharyya S., Mahoney M.C., Conant E.F., Bassett L.W., Pisano E.D. (2011). Use of BI-RADS 3–probably benign category in the American College of Radiology imaging network digital mammographic imaging screening trial. Radiology.

[48] Sickles E.A. (1999). Probably benign breast lesions: When should follow-up be recommended and what is the optimal follow-up protocol?. Radiology.

[49] Flowers C.I., O’Donoghue C., Moore D., Goss A., Kim D., Kim J.-H., Elias S.G., Fridland J., Esserman L.J. (2013). Reducing false-positive biopsies: A pilot study to reduce benign biopsy rates for BI-RADS 4A/B assessments through testing risk stratification and new thresholds for intervention. Breast Cancer Res. Treat..

[50] Brennan P.C., Ganesan A., Eckstein M.P., Ekpo E.U., Tapia K., Mello-Thoms C., Lewis S., Juni M.Z. (2019). Benefits of Independent Double Reading in Digital Mammography: A Theoretical Evaluation of All Possible Pairing Methodologies. Acad. Radiol..

