# Supplementary material for: Diagnostic Efficacy across Dense and Non-Dense Breasts during Digital Breast Tomosynthesis and Ultrasound Assessment for Recalled Women
Source: Diagnostics (Basel). 2022 Jun 16;12(6):1477. doi: 10.3390/diagnostics12061477 (PMC9222054; doi:10.3390/diagnostics12061477)
Supplement: Supplementary file 1 [file diagnostics-12-01477-s001.zip › diagnostics-1767166-supplementary.pdf]

diagnostics

Supplementary Material

Table S1. The agreement of RANZCR breast lesion classifications between DBT and DM spot views on 219 lesions.

		DBT					
	**RANZCR**	**1**	**2**	**3**	4	5	total
**DM spot views**	1	2	0	0	0	0	2
	2	1	1	0	0	0	2
	3	19	0	78	23	1	121
	4	2	1	7	42	7	59
	5	0	0	5	10	20	35
	total	24	2	90	75	28	219

**DBT vs DM spot views**	**Cohen's Kappa for 2 Raters**
Subjects	219
Raters	2
Agreement %	66
Kappa	0.49
z	11.8
p-value	< .001

Kappa: the estimation of weighted kappa (0-1); Agreement %: the relative observed agreement among raters; z: Estimates the asymptomatic standard error assuming the null hypothesis that weighted kappa is zero; DM: digital mammography; DBT: digital breast tomosynthesis.
